# Evidence for a Relationship between VEGF and BMI Independent of Insulin Sensitivity by Glucose Clamp Procedure in a Homogenous Group Healthy Young Men

**DOI:** 10.1371/journal.pone.0012610

**Published:** 2010-09-07

**Authors:** Michaela Loebig, Johanna Klement, André Schmoller, Simone Betz, Nicole Heuck, Ulrich Schweiger, Achim Peters, Bernd Schultes, Kerstin M. Oltmanns

**Affiliations:** 1 Department of Psychiatry and Psychotherapy, University of Luebeck, Luebeck, Germany; 2 Department of Internal Medicine I, University of Luebeck, Luebeck, Germany; 3 Department of Interdisciplinary Obesity Center, Kantonsspital St. Gallen, Rorschach, Switzerland; Karolinska Institutet, Sweden

## Abstract

**Background:**

This is the first study to experimentally explore the direct relationship between circulating VEGF levels and body mass index (BMI) as well as to unravel the role of insulin sensitivity in this context under standardized glucose clamp conditions as the methodical gold-standard. In order to control for known influencing factors such as gender, medication, and arterial hypertension, we examined a highly homogeneous group of young male subjects. Moreover, to encompass also subjects beyond the normal BMI range, low weight and obese participants were additionally included and stress hormones as a main regulator of VEGF were assessed.

**Methodology/Principal Findings:**

Under euglycemic clamp conditions, VEGF was measured in 15 normal weight (BMI 20–25 kg/m^2^), 15 low weight (BMI<20 kg/m^2^), and 15 obese (BMI>30 kg/m^2^) male subjects aged 18–30 years and the insulin sensitivity index (ISI) was calculated. Since stress axis activation promotes VEGF secretion, concentrations of ACTH, cortisol, and catecholamines were monitored. Despite of comparable ACTH (*P = 0.145*), cortisol (*P = 0.840*), and norepinephrine (*P = 0.065*) levels, VEGF concentrations differed significantly between BMI-groups (*P = 0.008*) with higher concentrations in obese subjects as compared to normal weight (*P = 0.061*) and low weight subjects (*P = 0.002*). *Pearson*'s correlation analysis revealed a positive relationship between BMI and VEGF levels (*r = 0.407*; *P = 0.010*) but no correlation of VEGF with ISI (*r = 0.224*; *P = 0.175*).

**Conclusions/Significance:**

Our data demonstrate a positive correlation between concentrations of circulating VEGF levels and BMI in healthy male subjects under highly controlled conditions. This relationship which is apparently disconnected from insulin sensitivity may be part of some pathogenetic mechanisms underlying obesity and type 2 diabetes.

## Introduction

Vascular endothelial growth factor (VEGF) is a cytokine known to increase vascular permeability and vasodilatation [1]. Moreover, there is evidence that VEGF is involved in the pathogenesis of cancer, arteriosclerosis, obesity, and diabetes mellitus-related complications such as diabetic retinopathy [1]–[3]. The synthesis and secretion of VEGF is affected by several variables *in vitro*, VEGF is up-regulated by hypoxia as well as by hyper- and hypoglycemia [4]–[6]. In contrast, circulating VEGF concentrations decrease upon hypoxia and increase upon hypoglycemia *in vivo* [7]–[9]. Among various sides within the organism, also adipose tissue as an endocrine organ produces VEGF in a considerable amount [10], [11]. Previous studies indicate that overweight and obese individuals display elevated serum VEGF levels [3], [12]–[14]. Recent data from two cross-sectional observational studies refer to a statistical BMI dependence of VEGF levels in normal weight subjects within a wide variance of male and female participants at different metabolic states [15], [16]. However, known influencing factors such as fasting blood glucose levels, gender, smoking, elevated blood lipids, or activated stress axes may influence plasma VEGF concentrations [16], [17] and have not been considered yet. In type 2 diabetes mellitus whose pathogenesis is tightly linked to increased body mass index [18], it has been shown that the insulin sensitivity is decreased [19] suggesting a direct negative relationship between VEGF concentrations and insulin sensitivity.

In order to further explore the indicated relationship between plasma VEGF concentrations and BMI as well as the role of insulin sensitivity in this context, we experimentally tested a highly homogeneous group of young healthy male subjects under the metabolically controlled conditions of a glucose clamp as the gold standard to investigate insulin sensitivity [20]. Moreover, this method allows for the exclusion of blood glucose variances as bias. Also, to additionally check if this relationship is also confirmed in subjects beyond the normal BMI range, we included low weight and obese subjects. We hypothesized a direct relationship between plasma VEGF levels and body mass index (BMI) comprising all BMI groups.

Since BMI and insulin sensitivity are known to be inversely related [21] we moreover assumed a negative correlation between VEGF levels and the insulin sensitivity index (ISI) which was determined by hyperinsulinemic-euglycemic clamp technique. Further, we monitored concentrations of catecholamines, cortisol, and ACTH to control for the well known influence of stress hormones on VEGF concentrations and insulin sensitivity [22]–[24]. Considering the known release of VEGF by thrombocytes [25], we measured VEGF levels in plasma samples.

## Results

### Glucose metabolism and insulin sensitivity

Table 1 shows main measures of the glucose clamp procedure. The three subject groups showed no difference in fasting glucose (*P = 0.304*) and fasting insulin (*P = 0.160*) levels. During the euglycemic clamp, mean glucose levels were comparable between groups (*P = 0.102*). Likewise, glucose infusion rates did not differ significantly between the three body mass groups (*P = 0.074*). As expected, obese subjects revealed higher insulin and c-peptide concentrations than normal and low weight subjects (*insulin P<0.001*; *c-peptide P = 0.002 for group comparison*). M-values (*P = 0.011*) and ISI values (*P = 0.01*) reflect the known negative relationship between BMI and insulin sensitivity (Table 1).

**Table 1:** Relevant basic characteristics of the participants, measurements of the glucose clamp procedure, and stress hormone levels.

Category	Obese subjects	Normal weight subjects	Low weight subjects	P-value
Age (years)	27.80±1.07	26.27±0.96	22.53±0.68	<0.001*
Body height (m)	1.83±0.18	1.88±0.15	1.80±0.002	0.071
Body weight (kg)	112.81±3.7	77.12±1.3	63.6±1.5	<0.001*
BMI (kg/m^2^)	33.81±1.1	21.86±0.3	18.97±0.2	<0.001*
Glucose (mmol/l)	5.0±0.1	4.8±0.2	5.1±0.1	0.102
Glucose infusion rates (ml/h)	150.6±20.7	182.4±19.3	123.9±12.2	0.074
Insulin (µU/ml)	87.8±7.4	53.4±3.9	50.0±6.1	<0.001*
C-peptide (ng/ml)	3.4±0.4	1.8±0.3	1.7±0.3	0.002*
M value † (mg×kg^−1^×min^−1^)	7.20±1.02	12.36±1.35	9.34±1.01	0.011*
ISI †(100×mg×l×kg^−1^×min^−1^×mU^−1^)	6.09±1.09	17.82±3.87	13.43±1.57	0.010*
ACTH (pg/ml)	25.5±3.0	21.6±3.6	17.2±1.3	0.145
Cortisol (µg/ml)	10.7±0.8	10.7±0.9	10.6±0.7	0.840
Epinephrine(pg/ml)	20.9±1.7	36.2±5.2	39.8±6.5	0.041*
Norepinephrine (pg/ml)	263.4±26.4	272.7±25.1	199.6±18.9	0.065

Indicated are mean values ± SEM; P-values are based on ANOVA analysis.

*mark significant group differences.

† M values and ISI values were calculated as described by Eriksson et al. [32].

### Serum concentrations of ACTH, cortisol, and catecholamines

Concentrations of ACTH, cortisol, and catecholamines are summarized in Table 1. The three body mass groups did not differ with respect to ACTH (*P = 0.145*), norepinephrine (*P = 0.065*), and cortisol (*P = 0.840 for group comparison*) concentrations. Regarding epinephrine concentrations, we found a significant group main effect (*P = 0.041*) which was mainly based on lower levels in obese participants than in normal weight controls (*P = 0.027*). Also, epinephrine concentrations measured in the obese subject group were significantly lower than values of the low weight subjects (*P = 0.026 for group main effect*).

### Plasma VEGF concentrations

**Figure 1:**
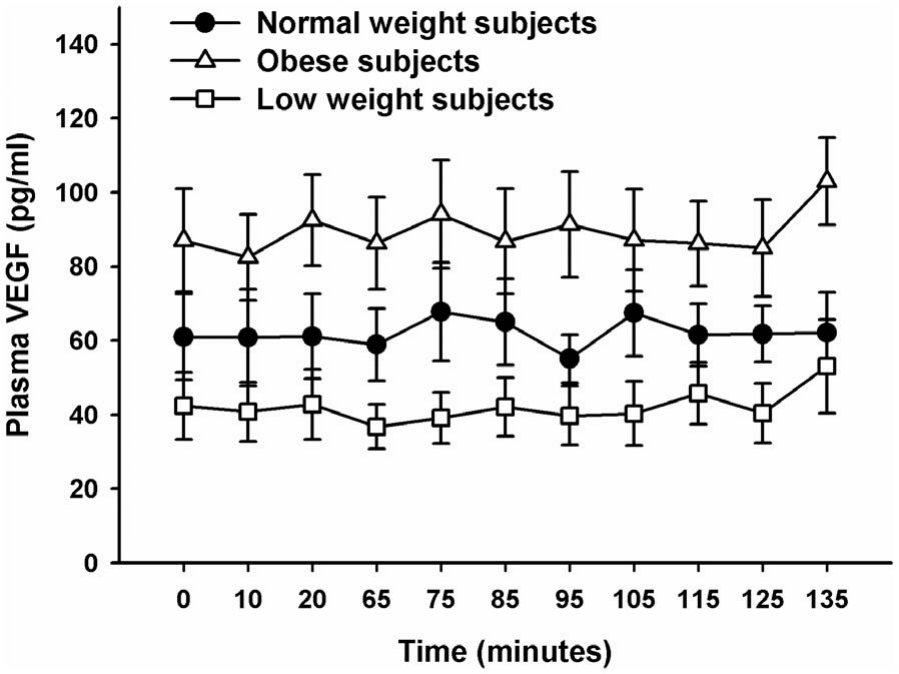
Plasma VEGF concentrations during the experiment. Plasma VEGF concentrations (mean values ± SEM) at baseline and during the euglycemic clamp procedure. Time point 65 until time point 105 was included in the calculation of the ISI values.

Figure 1 shows VEGF concentrations in normal weight, obese and low weight subjects under euglycemic clamp conditions. Groups differed significantly (*P = 0.008 for group main effect*) showing a trend for lower VEGF concentrations in normal weight than in obese subjects (*P = 0.061*) and clearly higher VEGF levels in obese as compared with low weight subjects (*P = 0.002*). When comparing low weight subjects with normal weight controls, analysis failed to reach significance (*P = 0.156*).

**Figure 2:**
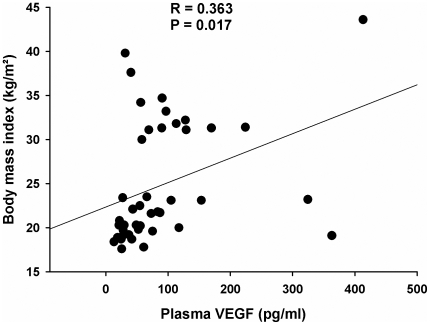
Correlation between plasma VEGF and BMI. Correlation of mean plasma VEGF levels (including all time points) with body mass index by Pearson's bivariate correlation analysis.

Notwithstanding, after merging all VEGF data of the three subject groups, we found a clear positive correlation between VEGF concentrations and BMI (*r = 0.363*; *P = 0.017 for group main effect*; *Fig. 2*). In contrast to our hypothesis, however, there was no correlation between VEGF levels and ISI (*r = 0.175*) despite of a clear inverse correlation between ISI and BMI (*r = −0.428; P = 0.007*; Table 1).

Correlation analyses between VEGF levels and concentrations of blood glucose (*P = 0.286*), ACTH (*P = 0.191*), cortisol (*P = 0.692*), epinephrine (*P = 0.745*), norepinephrine (*P = 0.093*), c-peptide (*P = 0.167*), or insulin (*P = 0.055*) revealed non-significant results.

## Discussion

Our data demonstrate a positive correlation between plasma VEGF concentrations and BMI over a large range of BMI between 17.6 kg/m^2^ and 43.6 kg/m^2^. Discrete group comparisons revealed significantly higher concentrations of plasma VEGF in obese subjects as compared with normal and low weight participants. However, comparing VEGF levels in normal and low weight participants did not reveal a significant difference. This lacking effect may be explained by too subtle BMI differences between the two body mass groups which was originated from the limited number of healthy male subjects with a BMI below 18.5 being considered as underweight (by WHO criteria). Our findings, however, are in line with data by Wada et al. who found by multiple regression analyses within a group of moderately overweight subjects with a metabolic syndrome that the independent determinants of VEGF were the body mass index and blood pressure [15]. Interestingly, in this study comparison of plasma VEGF concentrations between overweight subjects with a metabolic syndrome and a considerable number of normal weight healthy controls did not reach significance which casted some doubt on a direct relationship between circulating VEGF and BMI; a result which can probably be explained by the inhomogeneity of the included subject groups. In contrast, our data are also in accordance with results by Sandhofer et al. who observed in a population based cross-sectional study comprising slightly overweight male and female participants of different age only a weak correlation between plasma VEGF and BMI and drew the conclusion that circulating VEGF levels have only a minor impact on the development of atherosclerosis [16]. In this context, it is interesting that Randeva et al. could show in a considerable cohort of individuals that VEGF correlates significantly with the waist-to-hip ratio [26], a measure that, in turn, is suggested to be a better marker of subclinical arteriosclerosis than BMI. However, also two further studies indicated a relationship between BMI and plasma VEGF concentrations in healthy young men and women [3], [12] but did not take into consideration that gender influences VEGF concentrations significantly [3]. None of these previous studies encompassed also obese and low weight subjects which clearly strengthens the validity of our study. Note, since VEGF comprises a whole family of proteins (VEGF-A-F) displaying various characteristics, one must mention at this point that all mentioned studies in this context determined the same specific molecule, i.e. VEGF-A, using the same kits which allows for comparison between the studies.

Surprisingly, we did not find the assumed negative correlation between ISI and VEGF concentrations as assessed for the first time by standardized glucose clamp procedure although we could confirm the well known inverse relationship between BMI and insulin sensitivity [27]. This fact argues for a disconnection of insulin sensitivity from the relationship between VEGF and BMI in our study being apparently linked to other factors. In diabetic patients, increased VEGF concentrations are assumed to be caused by poor glycemic control [28]. In our study, we did not find a correlation between parameters of the glucose metabolism and VEGF levels but we could show risen serum insulin concentrations in the obese group as compared with the other groups which may, on the long term, lead to glycemic dysregulation. The influence of insulin per se on plasma VEGF, however, is not clarified yet. Dandona et al. [29] found a suppression of VEGF levels due to high insulin concentrations in obese subjects, a result that contrasts to our data rather indicating a relationship between high insulin levels and increased circulating VEGF concentrations. Notwithstanding, the influence of insulin on circulating VEGF still remains to be further clarified. ISI values, however, are regarded as a valuable tool to investigate insulin resistance. In the obese men of our study, ISI values were low and insulin concentrations were increased confirming insulin resistance as a well known phenomenon in overweight subjects. Whatsoever, there was no indication for an association between ISI and plasma VEGF in our study.

When trying to explain the underlying causes why VEGF concentrations correlate with BMI, some confounding factors need to be considered. In order to exclude the known bias of activated stress hormonal systems, we concomitantly monitored circulating concentrations of catecholamines as well as ACTH and cortisol. Our data, however, do not confirm the assumed influence of stress hormones on VEGF levels. Neither BMI group comparisons nor correlation analyses with VEGF concentrations revealed significant effects of ACTH and cortisol in contrast to previous findings [30]. In foregoing studies, it has been shown that also catecholamines influence VEGF levels by inducing VEGF expression [31]. Our catecholamine measurements are not in line with these data because we did not find a correlation between VEGF and catecholamine concentrations. Moreover, our norepinephrine levels showed no differences between groups and epinephrine levels were even lower in obese subjects than in the other BMI groups. Thus, catecholamines do not appear to be involved in mediating the relationship between VEGF levels and BMI. Another potential confounder in our study may be age [16]. Our low weight subjects were significantly younger than normal weight and obese subjects. Notwithstanding, the age difference between normal weight and obese subjects did not reach significance and previous studies do not find any age related effects on plasma VEGF levels [3], [14]. Also, the narrow range of age in our study varying between 18 years and 30 years does not argue for age as an underlying factor of VEGF differences.

In summary, we have shown a positive correlation between circulating VEGF levels and body mass index which is independent of insulin sensitivity. Since the underlying mechanisms of these coherences and the role of VEGF in the pathogenesis of obesity currently remain unclear, further studies possibly also involving animal and in vitro approaches are desirable to answer open questions. Our results suggest that an obesity-associated VEGF rise could play a major role in the development of known type 2 diabetes related sequelae like diabetic retinopathie. Body weight may thus be a factor which is even more relevant in this context than the disturbed glucose metabolism itself.

## Methods

### Subjects

Three groups comprising 15 normal weight (BMI 20–25 kg/m^2^), 15 low weight (BMI<20 kg/m^2^), and 15 obese (BMI>30 kg/m^2^) male subjects aged 18–30 years were included. Relevant main characteristics are shown in Table 1. Exclusion criteria were acute or chronic illness incl. arterial hypertension, alcohol or drug abuse, smoking, competitive sports, shift work, exceptional physical or mental stress, and any kind of current medication. On the day preceding the tests, all subjects were requested to abstain from alcohol, not to perform any kind of exhausting physical activity, and to go to bed no later than 11:00 p.m. Each participant gave written informed consent and the study was approved by the ethics committee at the University of Luebeck.

### Experimental design

On the day of the experiment, subjects arrived at the medical research unit at 08:00 a.m. after an overnight fast of at least 12 hours. One cannula was inserted into a peripheral vein of the arm, while the second one was inserted into an antecubital vein of the contralateral arm. The first blood sample was taken at 8:30 a.m. to determine baseline concentrations of VEGF, ACTH, cortisol, insulin, c-peptide, glucose, and catecholamines. After this baseline period, an insulin bolus (H-insulin, Hoechst, Frankfurt, Germany) was infused with a rate of 5 mU×min^−1^×kg^−1^ for 2 min followed by a continuous infusion rate of 1.5 mU×min^−1^×kg^−1^ for the next 100 min. A 20% dextrose solution was infused simultaneously at a variable rate to maintain a euglycemic plasma glucose level of 5.5 mmol/l. This euglycemic plateau was stable after 55 minutes of insulin infusion and maintained for another 45 minutes. Blood glucose concentrations were monitored at 5 min intervals during the experiments (B-Glucose-Data-Management, HemoCue GmbH, Grossostheim, Germany). Hormonal blood samples were drawn every 10 minutes throughout the experiment.

Blood was centrifuged within 5 min after withdrawal and serum as well as plasma was kept at −72°C until assay. At 10:20 a.m., infusion of insulin was stopped and the euglycemic state was reached after 30 minutes of glucose infusion.

### Assays

Plasma VEGF A samples were measured by ELISA (VEGF inter-assay coefficient of variation <8.8%, intra-assay CV 6.7%; R&D Systems, catalogue number SVE00, Mineapolis, MN). Serum ACTH, cortisol, c-peptide, and insulin concentrations were measured by commercial enzyme-linked immunoassays (all Immulite, DPC, Los Angeles, USA) with the following intra-assay and inter-assay coefficients of variation respectively: cortisol <5.8% and <6.3%, c-peptide <7.6% and <10.5%, insulin <5.2% and <6.1%, ACTH <6.1% and <9.4%. Plasma epinephrine and norepinephrine concentrations were measured by standard high-performance liquid chromatography with electrochemical detection (Recipe Chemicals+Instruments, Munich, Germany) with the following intra-assay and inter-assay coefficients of variation, respectively: epinephrine <7.6% and <4.2%, norepinephrine <6.7% and <5.3%.

### Statistical analysis

Values are presented as mean values ± standard error of mean (SEM). Statistical analyses were based on analysis of variance for repeated measurements (ANOVA) with the factors “time” (11 or 5 time points in case of catecholamine determination) and “group” (normal, obese, low weight subjects), followed by post hoc t-tests for pair wise comparisons. ISI was calculated as described by Eriksson et al. [32] and P-values were charged by oneway ANOVA. Correlations were performed by bivariate correlation analysis according to Pearson. A P-value<0.05 was considered significant.
